# Anandamide Protects HT22 Cells Exposed to Hydrogen Peroxide by Inhibiting CB1 Receptor-Mediated Type 2 NADPH Oxidase

**DOI:** 10.1155/2014/893516

**Published:** 2014-07-17

**Authors:** Ji Jia, Lei Ma, Mingchun Wu, Lei Zhang, Xiajing Zhang, Qian Zhai, Tao Jiang, Qiang Wang, Lize Xiong

**Affiliations:** ^1^Department of Anesthesiology, Xijing Hospital, Fourth Military Medical University, Xi'an 710032, China; ^2^Department of Anesthesiology, Xi'an No. 4 Hospital, Xi'an 710032, China

## Abstract

*Background.* Endogenous cannabinoid anandamide (AEA) protects neurons from oxidative injury in rodent models; however the mechanism of AEA-induced neuroprotection remains to be determined. Activation of neuronal NADPH oxidase 2 (Nox2) contributes to oxidative damage of the brain, and inhibition of Nox2 can attenuate cerebral oxidative stress. We aimed to determine whether the neuronal Nox2 was involved in protection mediated by AEA. *Methods.* The mouse hippocampal neuron cell line HT22 was exposed to hydrogen peroxide (H_2_O_2_) to mimic oxidative injury of neurons. The protective effect of AEA was assessed by measuring cell metabolic activity, apoptosis, lactate dehydrogenase (LDH) release, cellular morphology, intracellular reactive oxygen species (ROS), and antioxidant and oxidant levels and Nox2 expression. *Results.* HT22 cells exposed to H_2_O_2_ demonstrated morphological changes, decreased LDH release, reduced metabolic activity, increased levels of intracellular ROS and oxidized glutathione (GSSG), reduced levels of superoxide dismutase (SOD), and reduced glutathione (GSH) and increased expression of Nox2. AEA prevented these effects, a property abolished by simultaneous administration of CB1 antagonist AM251 or CB1-siRNA. *Conclusion.* Nox2 inhibition is involved in AEA-induced cytoprotection against oxidative stress through CB1 activation in HT22 cells.

## 1. Introduction

Oxidative stress is implicated in the pathology of many central nervous system (CNS) disorders, including Alzheimer's disease, Parkinson's disease, and ischemic stroke [1–3]. Hydrogen peroxide (H_2_O_2_) is produced at nearly every stage of the oxidative cycle and widely applied to induce oxidative stress* in vitro* [4]. H_2_O_2_-induced oxidative stress can cause lipid peroxidation, mitochondria injury, and DNA damage [5, 6].

NADPH oxidase (Nox) is a membrane-associated enzyme complex consisting of several subunits including NADPH oxidase 2 (Nox2). Activation of neuronal Nox2 contributes to oxidative damage of the CNS [7], and inhibition of Nox2 can attenuate cerebral oxidative stress injury [8]. We have previously demonstrated that inhibition of Nox2 reduced the damage induced by oxygen glucose-deprivation to a mouse hippocampal neuron cell line, HT22 [9].

Endogenous cannabinoid anandamide (N-arachidonoylethanolamine, AEA) mimics the bioactivity of Δ^9^-tetrahydrocannabinol (THC), the principal psychoactive component of marijuana [10]. There are two main cannabinoid receptors, CB1 and CB2 [11]. In the CNS, CB1 is mainly expressed in neurons, and CB2 in glial cells, such as microglia and astrocytes [11]. It was recently demonstrated in rodent models that AEA conferred neuroprotection by activating cannabinoid receptors. AEA could protect the newborn brain against excitotoxicity by activating CB1 [12] and attenuated cytotoxic edema caused by administration of Na^+^/K^+^-ATPase inhibitor [10]. We have previously reported that electroacupuncture pretreatment induces neuroprotection by stimulating release of AEA through a protein kinase C epsilon-mediated pathway [13]. However, the precise mechanism by which AEA mediated protection in the CNS remains undefined. The aim of this study was to determine whether AEA could protect HT22 cells against H_2_O_2_-induced injury and whether Nox2 was involved in the AEA-induced protection from oxidative stress via activation of CB1.

## 2. Materials and Methods

### 2.1. Materials

The HT22 cell line was a gift from Xuzhou Medical College (Xuzhou, China). The primary anti-CB1 antibody and primary anti-Nox2 antibody were purchased from Abcam Ltd. (Cambridge, UK), the primary anti-cleaved caspase-3 antibody was obtained from Santa Cruz (USA), and bovine serum albumin (BSA) and the cy3-labeled secondary antibody were purchased from Beijing Cowin Bioscience Co., Ltd. (Beijing, China). The AEA, AM251, Dulbecco's Modified Eagle Medium (DMEM), fetal bovine serum (FBS), apocynin, 3-(4,5-dimethyl-2-thiazolyl)-2,5-diphenyl-2-tetrazolium bromide (MTT), and dimethyl sulfoxide (DMSO) were purchased from Sigma-Aldrich (St. Louis, MO, USA). The 4′,6-diamidino-2-phenylindole (DAPI) and ROS Reagent kit were obtained from Beyotime (Nantong, China). The lactate dehydrogenase (LDH), superoxide dismutase (SOD), and reduced glutathione (GSH) and oxidized glutathione (GSSG) kits were purchased from Nanjing Jiancheng Bioengineering Institute (Nanjing, China).

### 2.2. Cell Culture

HT22 cells were cultured in DMEM with 10% FBS (v/v), 100 U/mL penicillin, and 100 *μ*g/mL streptomycin at 37°C in a humidified atmosphere containing 5% CO_2_ and 95% air. The medium was replaced every 2 days. When cell density reached roughly 70–80%, cells were exposed to the indicated drugs for 3 h. Then some evaluations were performed.

### 2.3. Experimental Protocols

**Figure 1:**
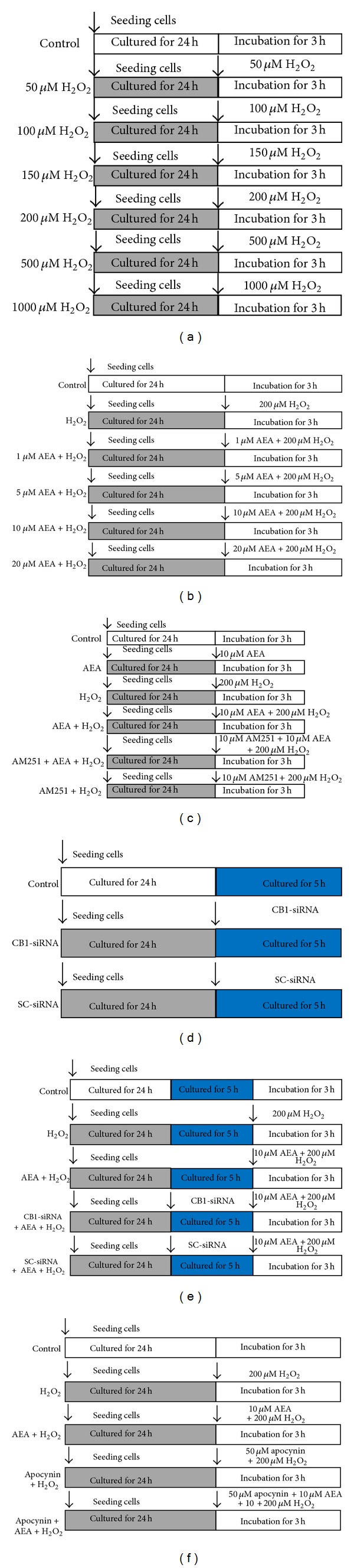
Experimental protocol diagram. (a) The HT22 cells were assigned into seven groups. The control cells were cultured in drug-free medium, and the other six groups were exposed to different concentrations of H_2_O_2_ for 3 h, ranging from 50 *μ*M to 1000 *μ*M. MTT assay was taken to determine the injury degree. (b) The cells were divided into six groups; except for control and H_2_O_2_ only groups, the other four groups were exposed to 200 *μ*M H_2_O_2_ plus different concentrations of AEA for 3 h. MTT assay was taken to evaluate the injury degree. (c) The cells were assigned into six groups, including control, AEA, H_2_O_2_, AEA + H_2_O_2_, AM251 + AEA + H_2_O_2_, and AM251 + H_2_O_2_ groups. After an incubation of 3 h, MTT assay, LDH release, and western blotting were taken to determine the roles of CB1 and Nox2 in AEA-induced protection. (d) The cells were divided into three groups, including control, CB1-siRNA, and SC-siRNA groups; after an incubation of 5 h, western blotting was used to evaluate the silencing rate of CB1 protein expression. (e) Then, the cells were divided into five groups, including control, H_2_O_2_, AEA + H_2_O_2_, CB1-siRNA + AEA + H_2_O_2_, and SC-siRNA + AEA + H_2_O_2_; the cell injury was evaluated by MTT and LDH release at 3 h after incubation, and ROS generation was evaluated by measuring fluorescence intensity. (f) The cells were divided into five groups, including control, H_2_O_2_, AEA + H_2_O_2_, apocynin + AEA + H_2_O_2_, and apocynin + AEA + H_2_O_2_; western blotting, MTT assay, and LDH release were taken to measure Nox2 expression and cell injury.

To find a suitable H_2_O_2_ concentration, the HT22 cells were assigned into seven groups (Figure 1(a)). Except for control group, the other six groups were exposed to different concentrations of H_2_O_2_ for 3 h, ranging from 50 *μ*M to 1000 *μ*M. Then MTT assay was taken to determine the injury degree of the cells. To find a suitable AEA concentration, the cells were divided into six groups. Except for control and H_2_O_2_ only groups, the other four groups were exposed to 200 *μ*M H_2_O_2_ plus different concentrations of AEA (Figure 1(b)). Then MTT assay was taken to evaluate the injury degree of the cells.

Then the cells were assigned into six groups, including control, AEA, H_2_O_2_, AEA + H_2_O_2_, AM251 + AEA + H_2_O_2_, and AM251 + H_2_O_2_ groups. After an incubation of 3 h, MTT assay, LDH release, and western blotting were taken to determine the roles of CB1 and Nox2 in AEA-induced protection (Figure 1(c)). To further investigate the role of CB1 in AEA-induced protection against H_2_O_2_ in HT22 cells, the HT22 cells were divided into three groups, including control, CB1-siRNA, and SC-siRNA groups; after an incubation of 5 h, western blotting was used to evaluate the silencing rate of CB1 protein expression (Figure 1(d)). Then, the cells were divided into five groups, including control, H_2_O_2_, AEA + H_2_O_2_, CB1-siRNA + AEA + H_2_O_2_, and SC-siRNA + AEA + H_2_O_2_. Then cells injury was evaluated by MTT and LDH release at 3 h after incubation, and ROS generation was evaluated by measuring fluorescence intensity (Figure 1(e)). Apocynin, a specific Nox inhibitor [14], was used to further investigate the role of Nox2 in AEA-induced protection against H_2_O_2_. The cells were divided into five groups, including control, H_2_O_2_, AEA + H_2_O_2_, apocynin + AEA + H_2_O_2_, and apocynin + AEA + H_2_O_2_; western blotting, MTT assay, and LDH release were used to measure Nox2 expression and cell injury (Figure 1(f)).

### 2.4. Cell Viability

HT22 cells were plated at a density of 1 × 10^4^ cells/well in 96-well plates. After treatment with different drugs for 3 h, cell injury was evaluated by MTT assay. 20 *μ*L of 5 mg/mL MTT solution was added to each well, and after 4 h incubation at 37°C the supernatant of each well was carefully removed. Then 150 *μ*L DMSO was added per well to solubilize the formazan product. The plate was then shaken for 10 min to ensure the formazan had completely dissolved. Absorbance at 490 nm was evaluated using a spectrophotometer (TECAN, CH).

### 2.5. LDH Release

HT22 cells were plated at a density of 2 × 10^4^ cells/well into a 24-well plate. After the treatments, the supernatants of each well were removed for assessment of LDH release, which was measured according to the manufacturer's instructions for the LDH assay kit. In brief, 100 *μ*L of cell-free supernatant, 250 *μ*L of buffer, and 50 *μ*L of coenzyme were mixed homogeneously and the supernatant was incubated with this reaction mixture for 15 min at 37°C. Next, 250 *μ*L of 2,4-dinitrophenylhydrazine was added into the mixture and incubated for an additional 15 min at 37°C in the dark. Finally, 2.5 mL of 400 mM of NaOH was added into the mixture to stop the reaction. After 3 min, the absorbance of the mixture was determined at 440 nm by spectrophotometry. The absorbance of the sample blank, standard, and standard blank was measured at the same time. LDH activity was calculated according to the following formula:

(1) LDH  activity  (U/L) =[sample  OD−sample  blank  ODstandard  OD−standard  blank  OD]  ×2×1000  U/L.

### 2.6. Apoptotic Rate

HT22 cells were seeded into a 6-well plate at a density of 1 × 10^5^ cells/well. After treatment with H_2_O_2_, AEA, and/or AM251, cells were centrifuged at 1000 rpm for 5 min. After two washes with ice-cold phosphate buffered saline (PBS), cells were resuspended in binding buffer at a density of 1 × 10^6^ cells/mL. And 5 *μ*L of fluorescein 5-isothiocyanate [2-(3,6-dihydroxy-9H-xanthen-9-yl)-5-isothiocyanatobenzoic acid] FITC-conjugated anti-annexin-V staining antibody and 2 *μ*L of propidium iodide (PI) solution were added to 100 *μ*L of the binding buffer. After thorough mixing, and 15 min incubation at room temperature in the dark, the apoptotic rate was assessed by flow cytometry (BD, USA).

### 2.7. Immunocytochemistry

HT22 cells were seeded into five confocal microscopy special dishes at a density of 2 × 10^4^ cells/dish. After 24 h, the cells were incubated with drug-free medium, 200 *μ*M H_2_O_2_, 10 *μ*M AEA + 200 *μ*M H_2_O_2_, 10 *μ*M AM251 + 10 *μ*M AEA + 200 H_2_O_2_, and 10 *μ*M AM251 + 200 *μ*M H_2_O_2_, respectively, for 3 h before the dishes were washed three times with PBS, then fixed with 4% paraformaldehyde solution for 1 h, and blocked with 50 mg BSA/mL in PBS for 30 min. Cells were incubated with primary anti-CB1 antibody (1 : 50) for 24 h at 4°C and then washed three times with PBS, before incubation with Cy3-labeled secondary antibody (1 : 200) for 1 h at room temperature. 200 *μ*L of DAPI staining solution was added into each dish for 5 min; then the dishes were washed thrice with PBS. CB1 expression was assessed by a confocal microscope (FV10i, Olympus, Japan) (excitation = 550 nm; emission = 570 nm).

### 2.8. Intracellular ROS

Intracellular ROS was detected by a ROS reagent kit utilizing the ability of intracellular ROS to oxidize nonfluorescent and colorless 2′,7′-dichlorofluorescin diacetate (DCFH-DA) into a fluorescent dichlorofluorescein (DCF). Cells were incubated with 10 *μ*M DCFH-DA at 37°C for 10 min and then rinsed twice with PBS, and ROS level was immediately assessed under a Leica DMI6000B, fluorescence microscope (Leica Microsystems GmbH, Wetzlar, Germany) (excitation = 488 nm and emission = 525 nm). Images were analyzed using Image Pro-Plus software (IPP 6.0, Media Cybernetics, Silver Spring, MD, USA).

### 2.9. Intracellular SOD, GSH, and GSSG

HT22 cells were seeded into 6-well plates at a density of 5 × 10^5^ cells/well. After treatment with H_2_O_2_, AEA, and/or AM251 (Figure 1(c)), cells were harvested and homogenized in 0.5 mL of 0.1 M phosphate buffer (pH 7.4). The mixture was centrifuged at 3000 rpm for 10 min at 4°C, and supernatants were used for SOD, GSH, and GSSG activity assessments with the corresponding reagent kits by spectrophotometry.

### 2.10. Short Interfering RNA

The sequence of mouse-CB1 short interfering RNA (CB1-siRNA, sc-39911) and scrambled short interfering RNA (SC-siRNA, sc-37007) were purchased from Santa Cruz Biotechnology. HT22 cells were seeded in 6-well plates at a density of 2 × 10^5^ cells/well in 2 mL DMEM without FBS. When the cell density reached 60–80% confluence, cells were washed with 2 mL siRNA Transfection Medium (sc-36868). CB1-siRNA and SC-siRNA were added to the cells and incubated for 5 h at 37°C before 1 mL of normal growth medium containing 20% FBS was added into each well without removing the transfection mixture. After an additional incubation of 24 h, cells were harvested.

### 2.11. Immunoblot Analysis

HT22 cells were lysed with modified RIPA-buffer containing a protease inhibitor-cocktail and 100 *μ*M phenylmethanesulfonyl fluoride on ice for 30 min. The total protein content was qualified by a bicinchoninic acid kit. Total protein lysates were subjected to 12% sodium dodecyl SDS-PAGE and transferred onto polyvinylidene difluoride membranes. Membranes were incubated with rabbit anti-mouse primary antibody (CB1, 1 : 1000; Nox2/gp91phox, 1 : 1000; Abcam, UK; cleaved caspase-3, 1 : 500; Santa Cruz, USA) in PBS with 0.1% Tween-20 overnight at 4°C and then incubated for 1 h at room temperature with anti-rabbit IgG. *β*-Actin, tubulin, and GADPH served as the control, respectively. Expression was visualized by enhanced chemiluminescence. The signal was quantified by densitometry by an immunoblotting detection system (Alpha Innotech, USA).

### 2.12. Real-Time PCR

Total RNA was isolated from HT22 cells with TRIzol reagent (Invitrogen, USA) according to the manufacturer's instructions. Total RNA (5 *μ*g) was used for first strand cDNA synthesis using the cDNA synthesis kit (TaKaRa, Japan). The PCR conditions for Nox2 were as follows: after initial denaturation at 95°C for 5 min, 40 cycles of 94°C for 30 s, 58°C for 30 s, and 72°C for 1 min were performed, followed by a 10 min extension at 72°C. The Nox2 RNA primers were as follows: 5′-CAAGATGGAGGTGGGACAGT-3′ (sense) and 5′-CAGGAGCAGAGGTCAGTGTG-3′ (antisense); and for *β*-actin, 5′-GAT GAG ATTGGC ATG GCT TT-3′ (sense) and 5′-GAG AA G TGGGGT GGC TT-3′ (antisense). Quantification of Nox2 mRNA was normalized to *β*-actin. The specificity of the PCR amplification products was assessed by dissociation melting curve analysis. Relative multiples of changes in mRNA expression were determined with the relative comparative threshold method [15].

### 2.13. Statistical Analysis

SPSS 11.0 (SPSS Inc, Chicago, IL) was used to conduct statistical analysis. Values were expressed as means ± standard deviation (SD). Results were compared by one-way ANOVA, followed by Tukey's Multiple Comparison Test. *P* < 0.05 was considered statistically significant.

## 3. Results

### 3.1. AEA Protected HT22 Cells Exposed to H_2_O_2_ in a Dose-Dependent Manner

**Figure 2:**
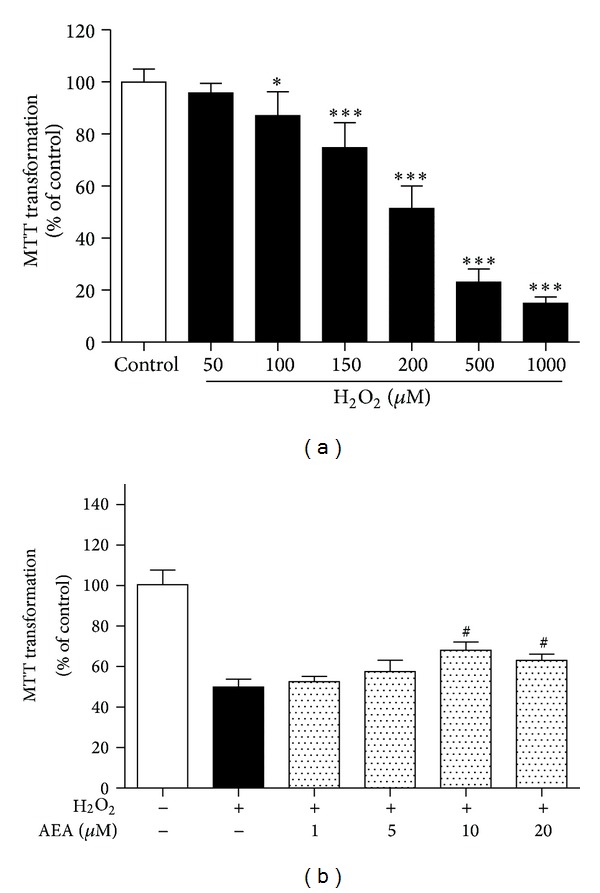
AEA increased the metabolic activity of HT22 cells exposed to H_2_O_2_ in a dose-dependent manner. (a) The correlation between the H_2_O_2_ concentration and cell metabolic activity. HT22 cells were exposed to different concentrations of H_2_O_2_ for 3 h (*n* = 8). (b) AEA increased the cell metabolic activity of HT22 cells exposed to 200 *μ*M H_2_O_2_ for 3 h (*n* = 8). Results are expressed as means ± SD, *P < 0.05, ***P < 0.001 versus the control (no H_2_O_2_, and no AEA), ^#^ *P* < 0.05 versus the cells exposed to H_2_O_2_ alone.

HT22 cells were exposed to H_2_O_2_ for 3 h, which decreased the cell metabolic activity in a dose-dependent manner. Exposure to 200 *μ*M H_2_O_2_ decreased the cell metabolic activity by roughly 50% (Figure 2(a)), and we used this condition for the subsequent experiments.

HT22 cells were exposed to 1 to 20 *μ*M of AEA in the presence of 200 *μ*M H_2_O_2_. 10 *μ*M and 20 *μ*M AEA significantly ameliorated the cytotoxic effect of H_2_O_2_ (Figure 2(b)). We used 10 *μ*M of AEA for subsequent experiments.

### 3.2. AEA Upregulated CB1 Expression in HT22 Cells

**Figure 3:**
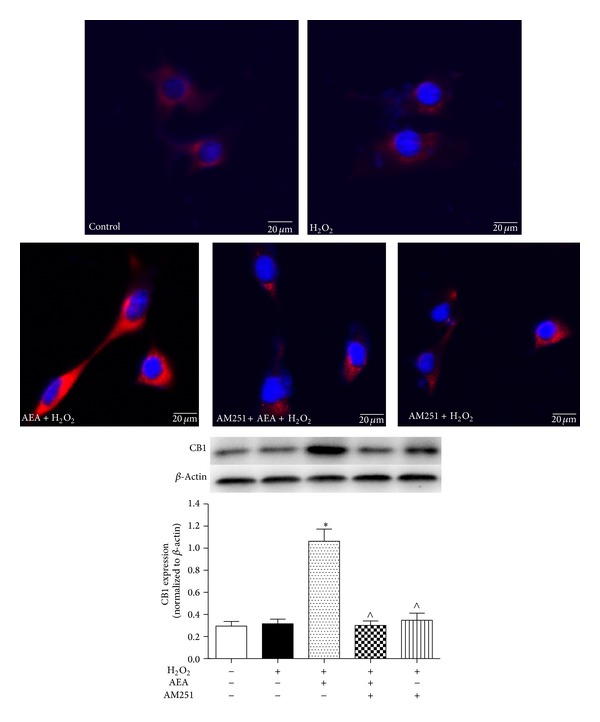
AEA upregulated the expression of CB1 in HT22 cells. Immunofluorescence staining and western blotting were used to investigate the AEA-induced effect on CB1 protein expression in HT22 cells. The cells were divided into five groups, Control: cells cultured in drug-free medium; H_2_O_2_: cells exposed to 200 *μ*M H_2_O_2 _for 3 h; AEA + H_2_O_2_: cells exposed to 10 *μ*M AEA plus 200 *μ*M H_2_O_2_ for 3 h; AM251 + AEA + H_2_O_2_: cells exposed to 10 *μ*M AEA plus 10 *μ*M CB1 antagonist AM251 at the presence of 200 *μ*M H_2_O_2 _for 3 h; AM251 + H_2_O_2_: cells exposed to 10 *μ*M AM251 plus 200 *μ*M H_2_O_2 _for 3 h. CB1 protein (red) was expressed in HT22 cells. AEA upregulated the expression of CB1 receptor; however CB1 antagonist AM251 reversed the CB1 upregulation in HT22 cells. Nuclei were counter-stained with DAPI (blue). Results are expressed as means ± SD (*n* = 4). *P < 0.05 versus control (no H_2_O_2_, no AEA, and no AM251), ^∧^ *P* < 0.05 versus the cells exposed to AEA plus H_2_O_2_. Bar = 20 *μ*m.

We used immunofluorescence and western blotting to assess whether AEA could up-regulate CB1 expression in HT22 cells. We observed CB1 staining in the cell membrane and cytoplasm of HT22 cells, consistent with a previous study [11]. Treatment with 10 *μ*M AEA induced a significant up-regulation of CB1 expression (*P* < 0.05), and the selective CB1 antagonist AM251 reversed the AEA-induced up-regulation of CB1 expression (Figure 3).

### 3.3. Protection of AEA against Oxidative Stress in HT22 Cells Involved CB1

**Figure 4:**
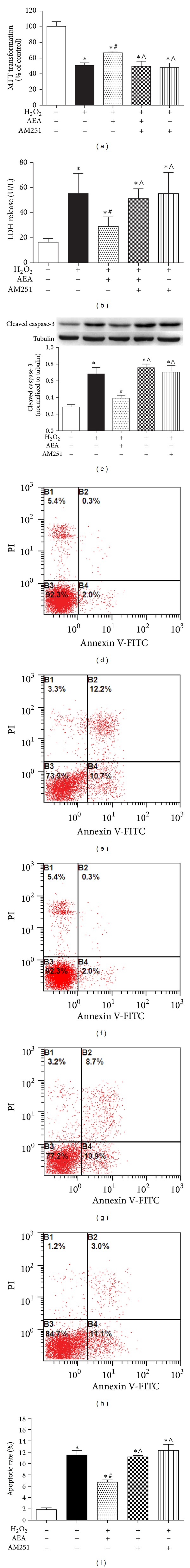
AEA protected HT22 cells exposed to H_2_O_2_ via CB1. (a) CB1 antagonist AM251 reversed AEA-induced protection on cell metabolic activity (*n* = 8). (b) AM251 reversed AEA-induced protection on LDH release (*n* = 6). (c) AM251 reversed AEA-induced reduction of cleaved caspase-3 expression (*n* = 4). (d)–(h) Apoptotic rates assessed by flow cytometry. (d) Control cells cultured in drug-free medium. (e) Cells exposed to 200 *μ*M H_2_O_2_ for 3 h. (f) Cells exposed to 10 *μ*M AEA plus 200 *μ*M H_2_O_2_ for 3 h. (g) Cells exposed to 10 *μ*M AEA plus 10 *μ*M AM251 in the presence of 200 *μ*M H_2_O_2_ for 3 h. (h) Cells exposed to CB1 antagonist AM251 of 10 *μ*M plus 200 *μ*M H_2_O_2_ for 3 h. (i) Statistical results of (c)–(g). Results are expressed as means ± SD (*n* = 4). *P < 0.05 versus the control (no H_2_O_2_, no AEA, and no AM251), ^#^ *P* < 0.05 versus the cells exposed to H_2_O_2_ alone, and ^∧^ *P* < 0.05 versus the cells exposed to AEA plus H_2_O_2_.

**Figure 5:**
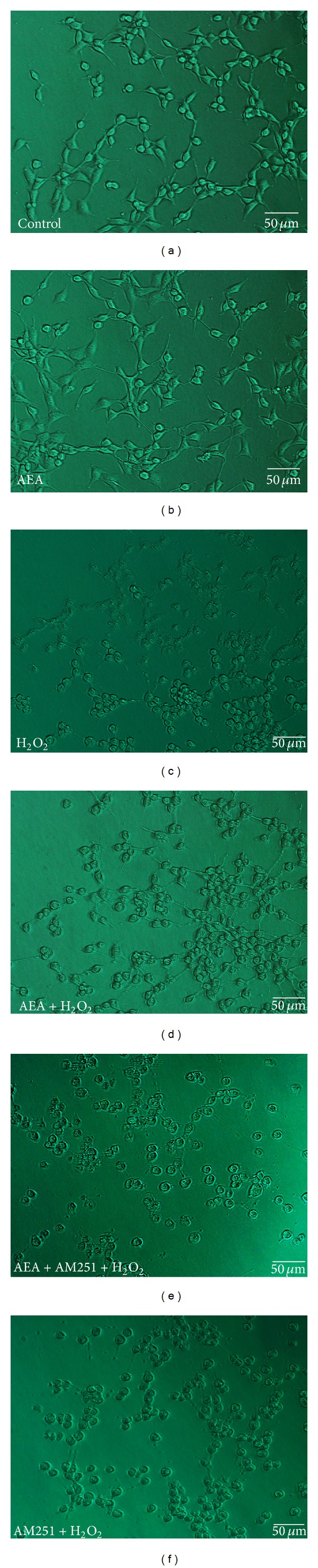
AEA ameliorated the morphology of HT22 cells exposed to H_2_O_2_ via CB1. (a) Control cells cultured in drug-free medium. (b) Cells exposed to 10 *μ*M AEA for 3 h. (c) Cells exposed to 200 *μ*M H_2_O_2_ for 3 h. (d) Cells exposed to 10 *μ*M AEA plus 200 *μ*M H_2_O_2_ for 3 h. (e) Cells exposed to 10 *μ*M AEA plus 10 *μ*M AM251 at the presence of 200 *μ*M H_2_O_2_ for 3 h. (f) Cells exposed to CB1 antagonist AM251 of 10 *μ*M plus 200 *μ*M H_2_O_2_ for 3 h. H_2_O_2_ markedly damaged the cell morphology and hindered the growth of neurites. AEA attenuated the H_2_O_2_-induced injury of HT22 cells whereas CB1 antagonist AM251 reversed the AEA-induced protective effect on cell morphology. Bar = 50 *μ*m.

In the absence of AEA, AM251 did not affect the cytotoxic impact of H_2_O_2_ (Figure 4(a)); however AM251 abolished the AEA-induced protection of HT22 cells, reducing the cell metabolic activity from 66.9 ± 2.4% to 49.5 ± 7.1% (*P* < 0.05). AM251 also reversed the influence of AEA on LDH release, increasing the LDH release from 29.1 ± 7.6 U/L to 51.2 ± 7.9 U/L (*P* < 0.05) (Figure 4(b)). We also evaluated cleaved caspase-3 expression and apoptotic rate by western blotting (Figure 4(c)) and flow cytometry (Figures 4(d)–4(i)), respectively, to assess the apoptosis of HT22 cells. AEA significantly decreased the expression of cleaved caspase-3 and the apoptotic rate of HT22 cells in response to H_2_O_2_ (*P* < 0.05). And AM251 abolished these effects caused by AEA. In addition, AEA ameliorated the changes in cellular morphology elicited by H_2_O_2_ and maintained the integrity of HT22 cells, and AM251 reversed this effect (Figure 5). These results indicated AEA protected HT22 cells from the damage caused by H_2_O_2_, and AM251 reversed this protection, suggesting that the protective effects of AEA may be mediated via CB1.

### 3.4. AEA Decreased Intracellular ROS and Maintained Intracellular Redox Status via CB1

**Figure 6:**
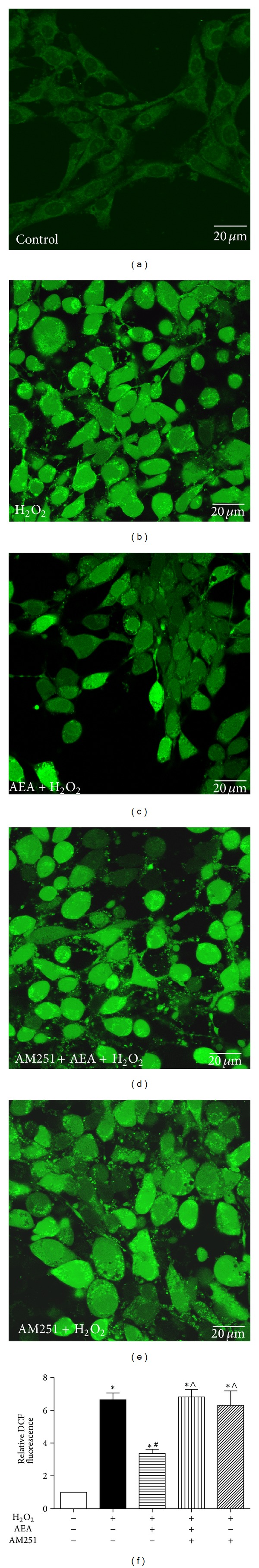
AEA decreased intracellular ROS generation via CB1. The intracellular ROS levels were assessed by ROS reagent kit. (a)–(e) indicate the fluorescence intensity of ROS. (a) Control cells cultured in drug-free medium. (b) Cells exposed to 200 *μ*M H_2_O_2_ for 3 h. (c) Cells exposed to 10 *μ*M AEA plus 200 *μ*M H_2_O_2_ for 3 h. (d) Cells exposed to 10 *μ*M AEA plus 10 *μ*M AM251 in the presence of 200 *μ*M H_2_O_2_ for 3 h. (e) Cells exposed to 10 *μ*M CB1 antagonist AM251 plus 200 *μ*M H_2_O_2_ for 3 h. (f) Statistical results of (a)–(e). Results are expressed as means ± SD (*n* = 6). *P < 0.05 versus the control (no H_2_O_2_, no AEA, and no AM251), ^#^ *P* < 0.05 versus the cells exposed to H_2_O_2_ alone, and ^∧^ *P* < 0.05 versus the cells exposed to AEA plus H_2_O_2_.

**Figure 7:**
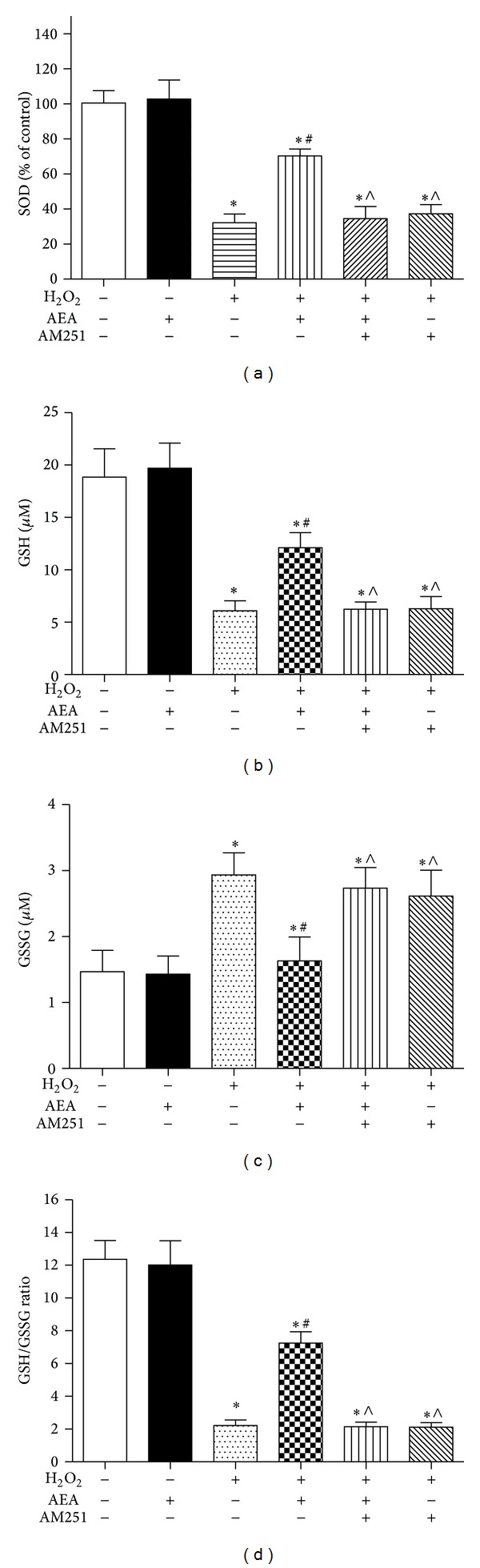
AEA increased intracellular SOD and ameliorated GSH/GSSG ratio. The cells were divided into six groups, Control: cells cultured in drug-free medium; AEA: cells exposed to 10 *μ*M AEA for 3 h; H_2_O_2_: cells exposed to 200 *μ*M H_2_O_2_ for 3 h; AEA + H_2_O_2_: cells exposed to 10 *μ*M AEA plus 200 *μ*M H_2_O_2_ for 3 h; AM251 + AEA + H_2_O_2_: cells exposed to 10 *μ*M AEA plus 10 *μ*M CB1 antagonist AM251 in the presence of 200 *μ*M H_2_O_2_ for 3 h; AM251 + H_2_O_2_: cells exposed to 10 *μ*M AM251 plus 200 *μ*M H_2_O_2_ for 3 h. The intracellular SOD, GSH, and GSSG levels were assessed by the corresponding reagent kit, and the GSH/GSSG ratio was calculated according to the GSH and GSSG levels. (a) Intracellular SOD level. (b) Intracellular GSH level. (c) Intracellular GSSG level. (d) Intracellular GSH/GSSG ratio. Results are expressed as means ± SD (*n* = 6). *P < 0.05 versus control (no H_2_O_2_, no AEA, and no AM251), ^#^ *P* < 0.05 versus the cells exposed to H_2_O_2_ alone, and ^∧^ *P* < 0.05 versus the cells exposed to AEA plus H_2_O_2_.

Exposure of HT22 cells to H_2_O_2_ led to accumulation of intracellular ROS, and simultaneous treatment with AEA markedly reduced the generation of ROS (Figure 6). SOD plays a vital role in protecting cells against oxidative injury. H_2_O_2_ treatment sharply decreased SOD activity in HT22 cells to 32.2 ± 5.0% (Figure 7(a)), and AEA restored SOD activity to 70.3 ± 4.0% of baseline (*P* < 0.05). GSH is also an important cellular antioxidant. H_2_O_2_ treatment sharply decreased GSH activity in HT22 cells from 18.8 ± 2.7 *μ*M to 6.1 ± 1.0 *μ*M (Figure 7(b)). Simultaneous application of AEA partially restored GSH levels to 12.1 ± 1.4 *μ*M (*P* < 0.05). GSSG levels were increased in response to H_2_O_2_ treatment from 1.5 ± 0.3 *μ*M to 2.9 ± 0.3 *μ*M (Figure 7(c)), and this effect was almost entirely abolished by AEA, which reduced GSSH levels to 1.6 ± 0.4 *μ*M (*P* < 0.05). The GSH/GSSG ratio was reduced from 12.3 ± 1.1 to 2.2 ± 0.3 by H_2_O_2_ treatment, and AEA partially restored this balance, increasing the ratio to 7.3 ± 0.7 (Figure 7(d)). The influences of AEA on intracellular ROS, SOD, GSH, GSSG, and GSH/GSSG ratio were abolished by the CB1 antagonist AM251, indicating that the antioxidative effects of AEA may be mediated via CB1 of HT22 cells.

### 3.5. CB1 Knockdown Reversed the Beneficial Effects of AEA on HT22 Cells Exposed to H_2_O_2_

**Figure 8:**
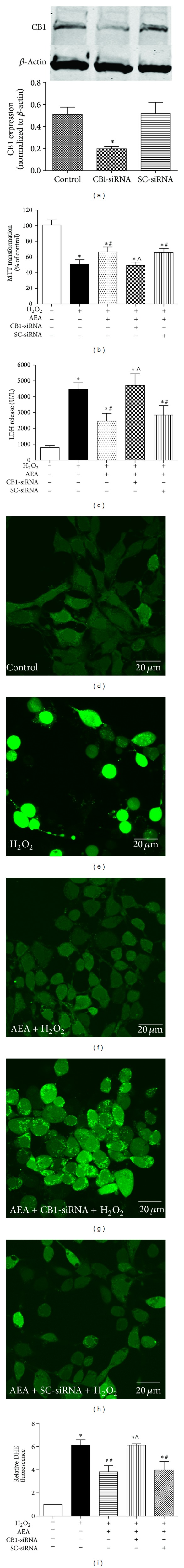
CB1-siRNA reversed AEA-induced protection against oxidative stress. The cells were divided into five groups, Control: cells cultured in drug-free medium; H_2_O_2_: cells exposed to 200 *μ*M H_2_O_2_ for 3 h; AEA + H_2_O_2_: cells exposed to 10 *μ*M AEA plus 200 *μ*M H_2_O_2_ for 3 h; CB1-siRNA + AEA + H_2_O_2_: cells incubated with CB1-siRNA for 5 h and then exposed to 10 *μ*M AEA plus 200 *μ*M H_2_O_2_ for 3 h; scrambled siRNA (SC-siRNA) + AEA + H_2_O_2_: cells incubated with SC-siRNA for 5 h and then exposed to 10 *μ*M AEA plus 200 *μ*M H_2_O_2_ for 3 h. CB1-siRNA abolished the AEA-induced protection against 200 *μ*M H_2_O_2_ in HT22 cells; SC-siRNA did not affect the protection. (a) CB1-siRNA significantly downregulated the expression of CB1, assessed by western blotting. (b) Cell metabolic activity, assessed by MTT (*n* = 8). (c) LDH release, assessed by reagent kit and spectrophotometry (*n* = 6). (d)–(h) The fluorescence intensity of ROS. (i) Statistical results of (d)–(h) (*n* = 6). Results are expressed as means ± SD, *P < 0.05 versus the control (no H_2_O_2_, no AEA, and no siRNA), ^#^ *P* < 0.05 versus the cells exposed to H_2_O_2_ alone, and ^∧^ *P* < 0.05 versus the cells exposed to AEA plus H_2_O_2_.

To further determine whether AEA-induced antioxidative ability was mediated by CB1 in HT22 cells, we used CB1-siRNA to knock down the expression of CB1. CB1-siRNA was effective in reducing the expression of CB1 (Figure 8(a)) and reversed AEA-induced cytoprotection, leading to a significant reduction of cell metabolic activity (Figure 8(b)), an increase of LDH release (Figure 8(c)), and a rise of intracellular ROS level (Figures 8(d)–8(i)).

### 3.6. Nox2 Was Involved in AEA-Induced Cytoprotection via CB1

**Figure 9:**
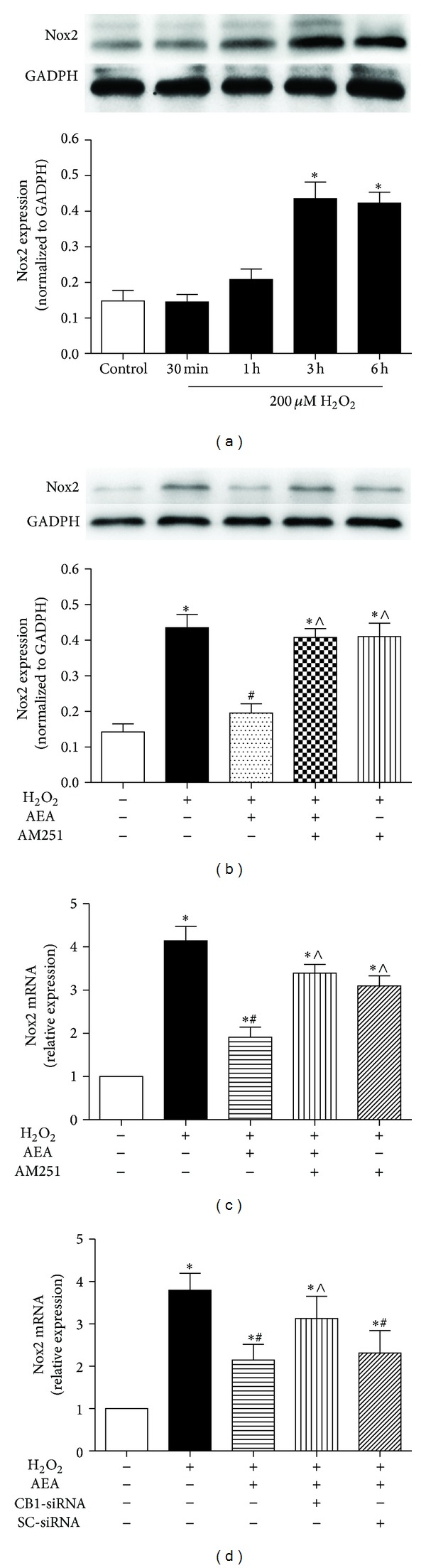
Nox2 expression was inhibited in the presence of AEA via CB1. (a) Nox2 expression was increased in HT22 cells exposed to H_2_O_2_ in a time-dependent manner. Then the cells were divided into five groups, Control: cells cultured in drug-free medium; H_2_O_2_: cells exposed to 200 *μ*M H_2_O_2_ for 3 h; AEA + H_2_O_2_: cells exposed to 10 *μ*M AEA plus 200 *μ*M H_2_O_2_ for 3 h; AM251 + AEA + H_2_O_2_: cells exposed to 10 *μ*M AEA plus 10 *μ*M CB1 antagonist AM251 in the presence of 200 *μ*M H_2_O_2_ for 3 h; AM251 + H_2_O_2_: cells exposed to 10 *μ*M AM251 plus 200 *μ*M H_2_O_2_ for 3 h. Nox2 protein expression (b) and mRNA transcription (c) were evaluated by western blotting and real-time PCR, respectively. (d) Incubation with CB1-siRNA for 5 h abolished the AEA-induced inhibition of Nox2 mRNA transcription. Results are expressed as means ± SD (*n* = 4). *P < 0.05 versus the control (no H_2_O_2_, no AEA, and no AM251 or siRNA), ^#^ *P* < 0.05 versus the cells exposed to H_2_O_2_ alone, and ^∧^ *P* < 0.05 versus the cells exposed to AEA plus H_2_O_2_.

The expression of Nox2 in HT22 cells was upregulated in the presence of 200 *μ*M H_2_O_2_ in a time-dependent manner (Figure 9(a)). AEA decreased the Nox2 protein expression (Figure 9(b)) and mRNA transcription (Figure 9(c)). However, CB1 antagonist AM251 or CB1-siRNA (Figure 9(d)) abolished AEA-induced influence on Nox2 protein expression and mRNA transcription, suggesting that the Nox2 may be involved in AEA-induced cytoprotection against H_2_O_2_ via CB1.

**Figure 10:**
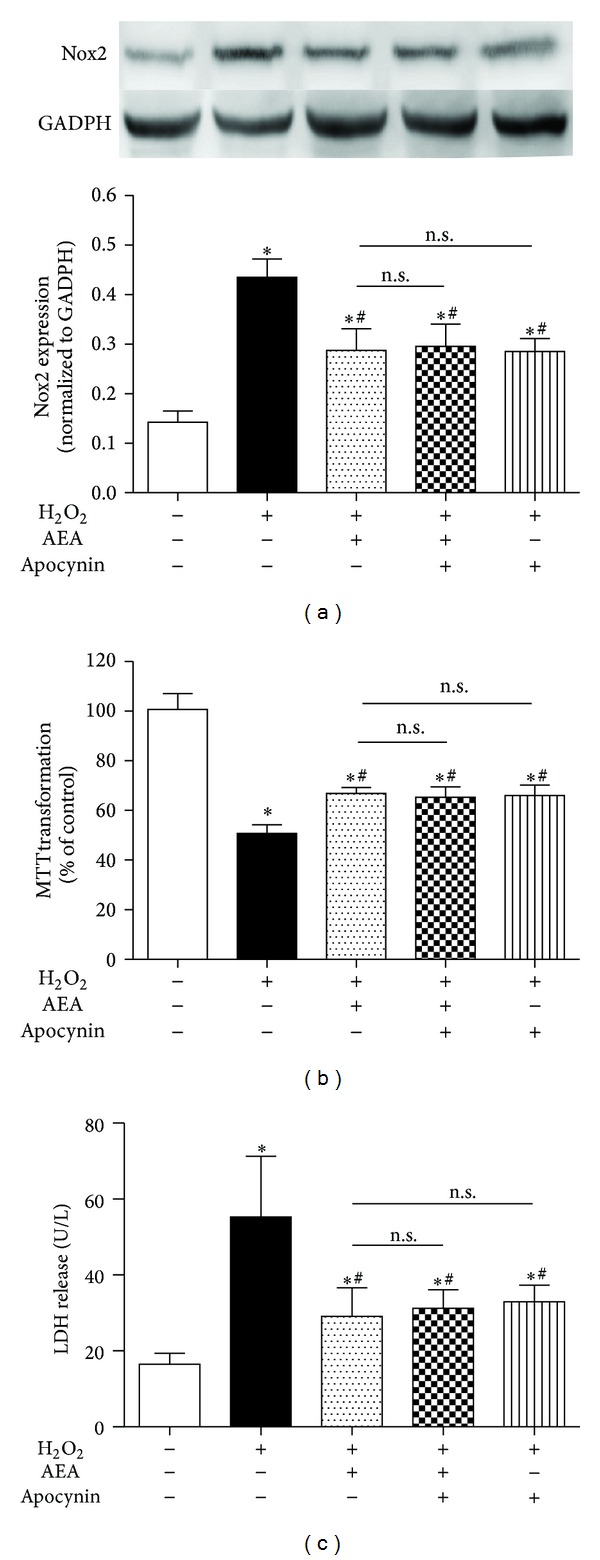
Nox inhibitor did not induce a more significant reduction of Nox2 expression than AEA alone. The cells were divided into five groups, Control: cells cultured in drug-free medium; H_2_O_2_: cells exposed to 200 *μ*M H_2_O_2_ for 3 h; AEA + H_2_O_2_: cells exposed to 10 *μ*M AEA plus 200 *μ*M H_2_O_2_ for 3 h; Apocynin + AEA + H_2_O_2_: cells exposed to 10 *μ*M AEA plus 50 *μ*M Nox inhibitor AM251 in the presence of 200 *μ*M H_2_O_2_ for 3 h; Apocynin + AEA + H_2_O_2_: cells exposed to 50 *μ*M apocynin plus 10 *μ*M AEA in the presence of 200 *μ*M H_2_O_2_ for 3 h. Nox2 protein expression (a) was evaluated by western blotting (*n* = 4). (b) Cell metabolic activity and (c) LDH release were determined by MTT (*n* = 8) and reagent kit (*n* = 6), respectively. Results are expressed as means ± S.D. *P < 0.05 versus the control (no H_2_O_2_, no AEA, and no apocynin), ^#^ *P* < 0.05 versus the cells exposed to H_2_O_2_ alone, n.s.: no significance.

To further investigate the role of Nox2 in AEA-induced protection in HT22 cells exposed to H_2_O_2_, we used apocynin, a specific Nox inhibitor. We found that the presence of 50 *μ*M apocynin decreased the expression of Nox2 protein significantly (Figure 10(a)), and there was no significance between AEA and apocynin alone on Nox2 expression in H_2_O_2_-treated HT22 cells. In addition, a combination of AEA and apocynin did not cause a more significant reduction of Nox2 expression than either AEA or apocynin used alone (*P* > 0.05). Similarly, we noticed a combination of AEA and apocynin did not induce a more significant increase of cell metabolic activity (Figure 10(b)) and reduction of LDH release (Figure 10(c)) than either AEA or apocynin alone (*P* > 0.05), indicating that Nox2 inhibition may be involved in AEA-induced cytoprotection against H_2_O_2_.

## 4. Discussion

In this study, we found that 10 *μ*M AEA treatment of a murine hippocampal neuron cell line, HT22, significantly improved cell injury, decreased apoptosis, and ameliorated the morphological changes induced by oxidative stress in the form of 200 *μ*M H_2_O_2_. Treatment with AEA reduced intracellular ROS and Nox2 expression in HT22 cells exposed to H_2_O_2_, and these effects were reversed by application of CB1 antagonist AM251 or CB1-siRNA. In addition, Nox inhibitor apocynin plus AEA did not induce a more significant downregulation of Nox2 or neuroprotection than apocynin or AEA used alone. These findings indicate that Nox2 inhibition is involved in AEA-induced neuroprotection against H_2_O_2_ through CB1 activation in HT22 cells.

Oxidative stress is involved in the pathophysiology of many CNS diseases [1–3]. Overaccumulation of intracellular ROS causes oxidative stress, which can damage cellular membranes, injure the mitochondria, and induce cell death. Thus enhancement of the cellular processes that suppress ROS generation or remove excess ROS may be effective in treating oxidative stress-induced diseases. Recently Nox proteins have been demonstrated to be major producers of ROS in CNS cells such as neurons, astrocytes, and microglia under pathophysiological conditions [16, 17]. Thus, inhibition of Nox proteins may present an effective mechanism to limit oxidative stress in the CNS. The Nox family includes Nox 1, 2, 3, 4, and 5, dual oxidase (DUAX) 1, and DUAX2 [18]. Nox2 appears to be the most important Nox in cerebral injury [19]. The infarct volume of Nox2 deficient mice is smaller than that of the wild-type and Nox2 deficient mice experience less blood-brain barrier injury than wild-type mice in a stroke model [20, 21].

Cannabinoids, such as AEA, appear to protect neurons against excitotoxicity, oxidative stress, and hypoxia through the activation of CB1 [12, 22]. Moldzio et al. reported that the cannabinoid THC protected dopaminergic neurons against 1-methyl-4-phenylpyridinium (MPP^+^) induced oxidative injury [23]. Chung et al. reported that cannabinoids WIN55, 212-2, and HU210 decreased lipopolysaccharide-induced activation of Nox in microglia [24]. We, therefore, hypothesize that inhibition of Nox may be involved in cannabinoid-induced protection from oxidative stress. In one of our previous studies, we observed that electroacupuncture induced neuroprotection against cerebral ischemia by increasing cerebral levels of the endogenous cannabinoid AEA [25]. However, AEA-mediated neuroprotection which is mediated via inhibition of Nox remained unknown. Thus, we investigated the protective effects of AEA against H_2_O_2_-induced neuronal injury.

The cannabinoid receptor CB1 is a G protein-coupled receptor present in both the cellular membrane and cytoplasm of neurons [26]. Nox2 is also mostly localized on the cellular membrane of neurons [19]. We found that antagonism of CB1 or silencing of CB1 expression reversed the AEA-induced inhibition of Nox2 protein expression and Nox2 mRNA transcription. In addition, Nox inhibitor apocynin alone or plus AEA did not induce a significant downregulation of Nox2 expression compared with AEA alone in the HT22 cells exposed to H_2_O_2_. Therefore, we inferred that AEA may activate CB1, which, in turn, inhibits Nox2 expression resulting in a reduction of cellular ROS.

Intracellular redox balance reflects the level of oxidative stress levels in cells and is crucial to cell function and survival. Accumulation of oxidants or overconsumption of antioxidants will damage cellular metabolism and even result in cell death [27]. In this study, we found that when in conditions of oxidative stress AEA raised intracellular SOD and GSH, reduced GSSG, and increased the GSH/GSSG ratio, and these effects were reversed by CB1 antagonist AM251, indicating that AEA could restore the balance of intracellular antioxidative and oxidative substances via CB1 receptor.

The ability of the endocannabinoid AEA to protect HT22 cells against H_2_O_2_-induced injury recommends AEA as a candidate therapy for oxidative stress-related neurological disorders. Endogenous agents have specific inactivation systems and therefore may run less risk of interfering with ongoing developmental profiles than artificial ligands. This is vital, as low levels of AEA can affect embryonic implantation, neural development, and suckling [28, 29]. Furthermore, cannabis use has been associated with the onset of schizophrenia [30, 31]. Exposure to synthetic cannabinoids WIN55, 212-2 caused disruption of learning and decreased emotional reactivity [32]. Thus interventions targeting the cannabinoid system need to be minimal during development [33] and endogenous agonists may be less deleterious.

However, further work must be done to determine the relevance of our findings* in vivo*. We have not fully elucidated the precise antioxidative mechanisms induced by AEA. In this study we investigated only the role of Nox2 in AEA efficacy, and whether other components of Nox are associated with AEA activities is not yet known.

## 5. Conclusions

This study provides evidence that the endocannabinoid AEA protects the mouse hippocampal neuron cell line HT22 against H_2_O_2_-induced oxidative injury through CB1-mediated inhibition of Nox2.
